# ROS-induced imbalance of the miR-34a-5p/SIRT1/p53 axis triggers chronic chondrocyte injury and inflammation

**DOI:** 10.1016/j.heliyon.2024.e31654

**Published:** 2024-05-22

**Authors:** Meng Zhou, Bi Liu, Hai-Ming Ye, Jia-Ning Hou, Yi-Cong Huang, Peng Zhang, Liang Gao, Hao-Tian Qin, Yi-Fei Yang, Hui Zeng, Bin Kang, Fei Yu, De-Li Wang, Ming Lei

**Affiliations:** a Department of Bone and Joint Surgery, Peking University Shenzhen Hospital, Shenzhen, 518036, Guangdong, China; b National and Local Joint Engineering Research Center for Orthopedic Biomaterials, Shenzhen, 518036, Guangdong, China; c Shenzhen Key Laboratory of Orthopaedic Diseases and Biomaterials Research, Shenzhen, 518036, Guangdong, China; d Department of Orthopedics and Traumatology, The Chinese University of Hong Kong, Hong Kong SAR, 999077, China; e Institute for Tissue Engineering and Regenerative Medicine, Faculty of Medicine, The Chinese University of Hong Kong, Hong Kong SAR, 999077, China; f Department of Orthopedics, Shenzhen People's Hospital, Shenzhen, 518020, Guangdong, China; g The Second Clinical Medical College, Jinan University, Shenzhen, 518020, Guangdong, China; h The First Affiliated Hospital, Southern University of Science and Technology, Shenzhen, 518020, Guangdong, China; i Department of General Surgery, Peking University Shenzhen Hospital, Shenzhen, 518036, Guangdong, China; j Department of Orthopedic Surgery, Affiliated Dongguan People's Hospital, Southern Medical University, Dongguan, 523000, China; k Center for Clinical Medicine, Huatuo Institute of Medical Innovation (HTIMI), Berlin, Germany

**Keywords:** miR-34a-5p/SIRT1/p53 axis, Chondrocyte injury and inflammation, Osteoarthritis, Reactive oxygen species, Apoptosis

## Abstract

Osteoarthritis is a chronic degenerative disease based on the degeneration and loss of articular cartilage. Inflammation and aging play an important role in the destruction of the extracellular matrix, in which microRNA (miRNA) is a key point, such as miRNA-34a-5p. Upregulation of miRNA-34a-5p was previously reported in a rat OA model, and its inhibition significantly suppressed interleukin (IL)-1β-induced apoptosis in rat chondrocytes. However, Oxidative stress caused by reactive oxygen species (ROS) can exacerbate the progression of miRNA regulated OA by mediating inflammatory processes. Thus, oxidative stress effects induced via *tert*-butyl hydroperoxide (tBHP) in human chondrocytes were assessed in the current research by evaluating mitochondrial ROS production, mitochondrial cyclooxygenase (COX) activity, and cell apoptosis. We also analyzed the activities of antioxidant enzymes including glutathione peroxidase (GSH-Px), catalase (CAT), and superoxide dismutase (SOD). Additionally, inflammatory factors, such as tumor necrosis factor (TNF)-α, interleukin (IL)-1β, IL-6, IL-8, and IL-24, which contribute to OA development, were detected by enzyme-linked immunosorbent assay (ELISA). The results of this study indicated that miR-34a-5p/silent information regulator 1 (SIRT1)/p53 axis was involved in the ROS-induced injury of human chondrocytes. Moreover, dual-luciferase assay revealed that SIRT1 expression was directly regulated by miR-34a-5p, indicating the presence of a positive feedback loop in the miR-34a-5p/SIRT1/p53 axis that plays an important role in cell survival. However, ROS disrupted the miR-34a-5p/SIRT1/p53 axis, leading to the development of OA, and articular injection of SIRT1 agonist, SRT1720, in a rat model of OA effectively ameliorated OA progression in a dose-dependent manner. Our study confirms that miRNA-34a-5p could participate in oxidative stress responses caused by ROS and further regulate the inflammatory process via the SIRT1/p53 signaling axis, ultimately affecting the onset of OA, thus providing a new treatment strategy for clinical treatment of OA.

## 1 Introduction

Osteoarthritis is a chronic degenerative disease characterized by degenerative changes in articular cartilage and secondary bone hyperplasia around the joints. Approximately 70 % of people over the age of 60 have radiological manifestations of OA, and this proportion gradually intensifies. For OA development, obesity and overuse of joints are the most remarkably variable risk factors [1,2]. Progressive articular cartilage loss is caused by molecular and mechanical processes. Molecular processes regulating OA are complex, but mainly include increased expression of extracellular matrix (ECM) catabolic enzymes, and pro-inflammatory cytokines [3], as well as reduced homeostatic process-related modification, such as autophagy. Those alterations eventually lead to cartilage degeneration and synovial inflammation, which can cause reduced mobility, stiffness, and joint pain [2]. There is no effective pharmacotherapy for OA to date. Morever, its pathogenesis is not entirely known owing to the hidden progress in the early stages and its complex etiology.

OA pathogenesis is significantly influenced by oxidative stress [4], and the impairment of cartilage and accompanying low-grade chronic inflammation are caused by the generation of ROS, comprising superoxide anion, nitric oxide, and hydrogen peroxide [5]. The oxidative metabolism of cells, comprising enzyme activity and mitochondrial respiration, produces free radicals containing oxygen molecules called ROS [6], and they are crucial for numerous cellular processes. The antioxidant defense system balances endogenous ROS generation under typical circumstances [7]. Nevertheless, ROS levels are elevated in numerous pathological manifestations, particularly in OA. ROS overproduction in chondrocytes causes DNA damage. Subsequently, their accumulation in cells trigger the activation of the metalloproteinases and aggrecanases and the degradation of the glycosaminoglycan and collagen, thus accelerating cartilage degradation [8]. Finally, oxidative stress induces OA chondrocyte apoptosis [9].

Intensified attempts have been made to comprehend the epigenetic causes of OA, particularly on the microRNA (miRNA)-mediated epigenetic regulation of OA progression. Small single-stranded non-coding RNA molecules (comprising nearly 22 nucleotides) called microRNAs are involved in post-transcriptional gene regulation and RNA silencing [10]. MiRNA expression patterns in OA patients differ remarkably from those of healthy people [11]. Moreover, some gene expressions regulated by miRNAs are responsible for articular homeostasis and chondrocyte function [12]., highlighting the miRNAs' significance in OA pathophysiology. The miRNA, miR-34a-5p is involved in p53-controlled tumor suppression and plays an integral role in p53-induced biological functions comprising senescence, apoptosis, cell cycle arrest, and proliferation. According to earlier research, patients with advanced OA (Kellgren/Lawrence [K/L] grade 3 or 4) have remarkably increased levels of miR-34a-5p in the synovial fluid [5] in comparison with the K/L grade 1 or 2 radiographic knee OA patients. Therefore, it is hypothesized that miR-34a-5p expression level is elevated in chondrocytes of advanced OA cartilage in comparison with the healthy cartilage and is associated with joint degeneration due to increased apoptosis. Previous study has shown that administration of miR-34a-5p antagonist alleviates chondrocyte death and cartilage degeneration in the joints of OA rat models. Nevertheless, further research is needed to completely understand the activity that miR-34a-5p plays during OA's treatment, comprising its role, signaling, and treatment potential [13].

Silent information regulator 1 (SIRT1) gene is the most widely studied type III deacetylase gene in mammals, closely related to aging, and also known as the longevity gene [14]. It can participate in the OA process by regulating oxidative stress, mitochondrial changes and protein modification [[15], [16], [17]]. The p53 gene is the most widely studied tumor suppressor gene in the human body, playing an important role in cell growth, apoptosis, and DNA repair regulation [[18], [19], [20]]. P53 can be regulated through AKT/mTOR, NF- κB signaling pathway affects the activity of chondrocytes [21,22]. Therefore, the SIRT1/p53 signaling axis plays an important role in the pathogenesis of oxidative stress mediated OA.

In this study, the function and treatment potential of targeting the miR-34a-5p/silent information regulator 1 (SIRT1)/p53 axis in knee OA pathogenesis was examined by subjecting the *in vivo* (rat OA models) and *in vitro* (human chondrocyte osteoarthritic [HC-OA] cells) models to miR-34a-5p mimic and miR-34a-5p antisense oligonucleotide (ASO) or SIRT1 agonist therapies, and preliminarily elucidated the possible mechanism of ROS induced oxidative stress in the pathogenesis of OA.

## 2 Materials and methods

### 2.1 Reagents and antibodies

TRIzol Plus RNA Purification Kit was obtained from Life Technologies (Carlsbad, CA, USA). SIRT1 agonist, SRT1720, was procured from MCE (Shanghai, China), Sigma-Aldrich (St. Louis, MO, USA) supplied *tert*-butyl hydroperoxide (tBHP) solution and Invitrogen™ provided 4′,6-diamidino-2-phenylindole (DAPI). The primary, as well as secondary antibodies that were employed in this research, are listed in Table 1.

**Table 1:** Primary antibodies used in this study.

Antibody	Host	Application	Source	Cat. No
Cleaved-Caspase3	Rabbit	WB(1:1000)/IHC(1:100)	CST*	#9661
Bcl-2	Rabbit	WB(1:1000)	Abclonal	A0208
Bax	Rabbit	WB(1:1000)	Abclonal	A0207
Collagen-2	Rabbit	WB(1:1000)/IHC(1:100)	Abcam	ab34712
IL-1β	Rabbit	WB(1:1000)	Abclonal	A19635
MMP13	Rabbit	WB(1:1000)	Abclonal	A16920
SIRT1	Rabbit	WB(1:1000)/IHC(1:100)	Abclonal	A19667
p53	Rabbit	WB(1:1000)	Abclonal	A19585
Acetyl-p53 (Lys379)	Rabbit	WB(1:1000)/IHC(1:100)	CST	#2570
Cytochrome C	Rabbit	WB(1:1000)	Abclonal	A0225
COX IV	Rabbit	WB(1:1000)	Abclonal	A6564
GAPDH	Mouse	WB(1:1000)	Abclonal	AC002

CST* Cell Signaling Technology.

### 2.2 Cell culture

HEK293T and HC-OA cell lines employed in this research were procured from the ATCC Company (Manassas, VA, USA) and Sigma-Aldrich (Burlington, MA, USA), respectively. HC-OA cells were derived from the human articular cartilage of OA patients. The cell lines were maintained in high glucose-DMEM (Invitrogen, Carlsbad, CA, USA) with 10 % fetal bovine serum (FBS) and 1 % penicillin‒streptomycin (10 ng/ml penicillin and 10 U/ml streptomycin) at 37 °C in a moistened environment containing 5 % CO_2_.

### 2.3 Cell viability assay

The cytotoxicity of tBHP on HC-OA was detected by CCK-8 according to the manufacturer's protocol. Cells were seeded into a 96-well plate and cultured for 24 h. Then, the chondrocytes were incubated with tBHP (0, 50, 100, 150, 200, 300 and 400 μM) for 24 h, followed by 100 μM of tBHP treatment on HC-OA for different durations (0 h, 2 h, 6 h, 12 h, 24 h, 36 h and 48 h). Each well of the 96-well plate was added with 100 μL of 10 % CCK-8 solution (Dojindo, Kumamoto, Japan) and incubated for 2 h at 37 °C. The absorbance of each well was detected at 450 nm and analyzed with a microplate reader (Thermo Fisher Scientific, Rockford, USA).

### 2.4 Flow cytometry analysis

Employing the Annexin V FITC/PI kit (BD Biosciences), the cell apoptosis rate was investigated following the instructions of the manufacturer. Briefly, two million cells subjected to different treatments were incubated for 30 min utilizing 5 μL of the activated Annexin V FITC/PI reagent at 37 °C and immediately examined employing the FL1/2 channel of a flow cytometer (BD Accuri C6).

### 2.5 Reverse transcription-quantitative polymerase chain reaction (RT-qPCR)

Extraction of total RNA from the chondrocyte cell line and rat knee joint cartilage tissues was performed with TRIzol Plus RNA Purification Kit (Life Technologies, Carlsbad, CA, USA) according to the manufacturer's instruction. The cDNAs were obtained through reverse transcription of total RNA utilizing a High Capacity cDNA Reverse Transcription Kit (Thermo Fisher Scientific, Waltham, MA, USA). The cDNA amplification primers are listed in Table 2. Glyceraldehyde 3-phosphate dehydrogenase (GAPDH) was used as an endogenous control. For miRNA isolation, miRcute miRNA Isolation Kit (DP501, Tiangen Biotech, Beijing, China) was employed as per the maker's guidelines, and reverse transcription was done for 100 μg miRNA employing miRcute Plus miRNA First-Strand cDNA Kit (KR211; Tiangen Biotech). The miRcute Plus miRNA qPCR Kit (FP411; Tiangen Biotech, Beijing, China) was used to quantify miRNA expression levels, and the relative fold change (2^−ΔΔCT^) was used to calculate relative gene expression. PCR amplification was performed on the DNA engine CFX96 Real-Time PCR Amplification System (CFX96 Touch; Bio-Rad Laboratories, Feldkirchen, Germany). All the experiments were conducted in triplicates.

**Table 2:** qPCR primers used in this study.

Target gene	Forward	Reverse
Homo-GAPDH	GTCTCCTCTGACTTCAACAGCG	ACCACCCTGTTGCTGTAGCCAA
hsa-miR-34a-5p	GGCAGTGTCTTAGCTGG	GAACATGTCTGCGTATCTC
Homo-SIRT1	TAGACACGCTGGAACAGGTTGC	CTCCTCGTACAGCTTCACAGTC
Rat-NF-κB	TTCAACATGGCAGACGACGA	TGCTCTAGTATTTGAAGGTATGGG
Rat-MMP13	TCCATCCCGAGACCTCATGT	CTCAAAGTGAACCGCAGCAC
Rat-ACAN	AGCCCTTGTCTGAATGGAGC	GTTGGTTTGGACGCCACTTC
nor-miR-34a-5p	TGGCAGTGTCTTAGCTGGTT	AACGTGCAGCACTTCTAGGG
Rat-GAPDH	GCATCTTCTTGTGCAGTGCC	GATGGTGATGGGTTTCCCGT

### 2.6 Transfection and infection of miR-34a-5p and anti-miR-34a-5p

All miRNAs used to regulate miR34a expression, including miR-34a-5p, mimic negative control, miR-34a-5p mimic, inhibitor negative control, and anti-miR-34a-5p (hsa-miR-34a-5p Inhibitor), were purchased from RiboBio (Guangzhou, China). Specifically, primary chondrocytes were cultured for 24 h on a 6-well plate (Costar 3516; Corning NY, USA) at a density of 2 × 10^5^ cells/well. Utilizing lipofectamine 3000 (Invitrogen, Carlsbad, CA, USA), transfection of the aforesaid miRNAs was performed for 72 h, after which cells were washed and lysed for protein analyses.

### 2.7 STR1720 administration

For *in vitro* experiments, HC-OA cells were cultured with different concentrations of SRT 1720 diluted in DMSO at different concentrations (0.01, 0.02, 0.05, 0.1, 0.2, 0.5, 1, 2, 5 μM) for 24 h. Cell viability was detected using CCK-8 assays. *In vivo*, SRT1720 was administrated by articular injection from the 8th-day post-MIA injection. Diluting the SRT1720 stock solution (liquefied in dimethyl sulfoxide [DMSO; Sigma-Aldrich]) by 0.9 % NaCl (1:1 v/v), the working solution was prepared. Thereafter, the OA rats were held firmly and 10 μL SRT1720 solution was slowly injected into their articular joint cavity at 0.1 and 0.5 mg/kg representing the low-dose and high-dose group, respectively. The sham control mice received an equivalent volume (10 μL) of DMSO and 0.9 % NaCl through injection. SRT1720 or DMSO was injected for 14 d, after which the rats were sacrificed.

### 2.8 Western blot (WB) analysis

Utilizing the primary antibodies enlisted in Table 1, WB was performed. Cell Signaling Technology supplied HRP-conjugated secondary antibodies (1:2000). SuperSignal™ West Pico Chemiluminescent Substrate kits of Thermo Scientific were utilized to enhance chemiluminescence for revealing immunoreactive bands, which were then visualized with the aid of the ChemiDoc MP Imaging System (Bio-Rad, USA). Scanning densitometry was employed to measure band intensities, and ImageJ software was utilized for analysis.

### 2.9 Mitochondrial complex activity assay

Isolation of mitochondria was done utilizing 5 × 10^6^ primary HC-OA cells with the aid of the Cell Mitochondria Isolation Kit (Beyotime Co., China), and utilizing Micro Mitochondrial Respiratory Chain Complex I to IV Activity Assay Kit (Solarbio, China), the mitochondrial complex I to IV activities were investigated following instructions of the manufacturer. Specifically, corresponding reaction buffers were combined with mitochondrial homogenates before being transferred to a quartz cuvette that had been preheated to 30 °C and promptly put in a spectrophotometer. The activity of the mitochondrial complex was articulated as nmol/min/mg protein.

### 2.10 Enzyme-linked immunosorbent assay (ELISA)

ELISA kits (KAC1211, EH2IL6, KAC1301, EH269RB, and BMS223-4; Invitrogen, USA) were used to determine the serum concentrations of tumor necrosis factor (TNF)-α, interleukin (IL)-1β, IL-6, IL-8, and IL-24, in accordance with the manufacturers' guidelines. With the aid of a microplate reader, the concentration of these cytokines was examined using the optimum density at 450 nm. The samples’ concentrations were determined by deriving the r value and curve equation.

### 2.11 Terminal deoxynucleotidyl transferase dUTP nick end labeling (TUNEL) assay

TUNEL–DAPI (Thermo Fisher Scientific, Waltham, MA, USA) double labeling was utilized to differentiate between apoptotic cells and non-apoptotic cells. HC-OA cells cultured on coverslips of glass were fixed with 4 % paraformaldehyde (PFA) and incubation was done utilizing TUNEL reaction mixture for 60 min. The cells were then examined using a fluorescent microscope. For each slide, six visual areas were chosen at random in order to count and compute the percentage of TUNEL-positive cells.

### 2.12 Immunofluorescent staining

HC-OA cells incubated on coverslips of glass were fixed utilizing 4 % PFA for 10 min and permeabilized utilizing 0.1 % Triton X-100 for 10 min. After 30 min incubation using 1 % BSA, to suppress nonspecific binding, overnight incubation of the cells was done at 4 °C with different primary antibodies. Thereafter, the incubation of cells was done at 37 °C utilizing an Alexa Fluor 594- or 488-labeled goat anti-rabbit (1:100; Invitrogen, Life Technologies, USA) secondary antibody for 1 h, and counterstaining was performed utilizing DAPI. A Zeiss 880 confocal microscope was employed to capture images (ZEISS, Germany).

### 2.13 Animals

The 8-week old male Sprague-Dawley rats utilized in the current research were purchased from Guangdong Medical Laboratory Animal Center (Guangzhou, China) and fed in the SPF-grade animal room of Peking University, Shenzhen Hospital Animal Center. Under a 12 h light/12 h darkness cycle and at 22 ± 1 °C, all animals weight ∼250 g at the beginning and were housed in cages with two rats per cage and were given access to standard food (full formula feed that had been irradiated for sterilization) as well as unlimited amounts of water. The Chinese Council on Animal Care's principles and regulations were strictly followed involving all of the study's animal trials. The Animal Ethical and Welfare Committee of Peking University, Shenzhen Hospital (Permit Number: 2020-301) granted its approval for the protocol, and measures were taken to minimize suffering.

### 2.14 Monosodium iodoacetate induction of OA

Adult Sprague-Dawley male rats (8 weeks old, weighing 250–260 g) underwent surgical induction of OA. Briefly, 24 rats were anesthetized with isoflurane, and a single intra-articular injection of 3.2 mg monosodium iodoacetate (MIA, Sigma, Poole, UK) was injected into the knee joint of the rats to induce OA. MIA was dissolved in 0.9 % sterile saline and administered in a volume of 40 μl using a 26-gauge needle. The control rats were administered an equivalent volume of saline through injection (n = 8). Daily observations were made for aberrant healing events at all surgery sites, including swelling, bleeding, dehiscence, redness, and drainage, among others.

### 2.15 Histological staining and immunohistochemistry analysis

The knee joints were collected after all the mice were sacrificed. Been fixed in 4 % paraformaldehyde for 72 h, the harvested joints were decalcified in 10 % (v/v) EDTA for 1 month. Specimens were then dehydrated, embedded in paraffin and cut into 5 μm slices. Slices were stained using hematoxylin-eosin (H&E) to assess cartilage destruction. After they were deparaffinized and rehydrated, sections were blocked in bovine serum albumin (BSA) containing 0.1 % Triton X-100 for 1 h for histochemistry. Sections were then incubated with antibody to collagen-2, acetyl-p53 and cleaved caspase 3 followed by HRP-conjugated secondary antibody。

### 2.16 Statistical analysis

All data are presented as the mean ± sem. Also, an unpaired Student's t-test was utilized for examining statistical significance, and *P* < 0.05 indicated a significance level.

## 3 Results

### 3.1 Effects of tBHP treatment on human chondrocytes

**Fig. 1:**
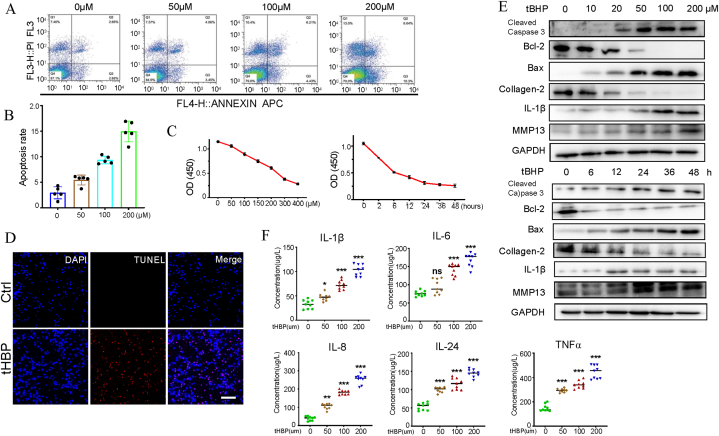
Effects of tBHP on HC-OA cells. (A, B) A concentration-dependent effect of tBHP on the chondrocyte apoptosis rate. Human chondrocytes were treated without/with different concentrations (50, 100 and 200 μM) of tBHP for 24 h. Cell apoptosis rates were determined by Annexin V-fluorescein isothiocyanate/propidium iodide staining and analyzed by flow cytometry. Values are expressed as percentages of cell viability at Cont. Data are the means ± sem of five independent experiments in duplicate. (C) A concentration- and time-dependent effect of tBHP on chondrocyte viability. Human chondrocytes were exposed to media containing different concentrations (0, 50, 100, 150, 200, 300 and 400 μM) of tBHP for 24 h and exposed to media containing 100 μM of tBHP for different durations (0 h, 2 h, 6 h, 12 h, 24 h, 36 h and 48 h). Cell viability was determined by a CCK8 assay. (D) Representative images of immunostaining for apoptotic (TUNEL-positive, red) chondrocytes. Nuclei were labeled with DAPI (blue). Scale bar represents 50 μm. (E) Western blot analysis for cleaved caspase-3, Bcl-2, Bax, Collagen-2, IL-1β and MMP13. (F) tBHP-mediated secretion of inflammatory factors in HC-OA cells. HC-OA cells were treated with the indicated concentrations (0, 50, 100, 200 μM) of tBHP for 24 h. The concentrations of TNF-α, IL-1β, IL-6 and IL-24 in the cell supernatants were measured by ELISAs. *P < 0.05, **P < 0.01 and ***P < 0.001 vs. Cont. (For interpretation of the references to colour in this figure legend, the reader is referred to the Web version of this article.)

For investigation of tBHP's toxic effect on human chondrocytes, we treated HC-OA cells with increasing amounts of tBHP (0, 50, 100, 200 μM) at different time points. The results revealed time- and dose-reliant effects of tBHP on chondrocyte apoptosis or viability according to the Annexin V FITC/PI assay (Fig. 1A and B) and the CCK8 assay (Fig. 1C), respectively. A dose of 100 μM tBHP for 24 h lead to a substantial elevation in the toxic effect of tBHP on the chondrocytes in comparison with the control, and this concentration was adopted in the subsequent experiments. Additionally, the TUNEL assay demonstrated a substantial increase in TUNEL-positive cells in the tBHP-treated cells in comparison with the control cells (Fig. 1D). Moreover, tBHP significantly stimulated caspase-3 and collagenase-3 (MMP-13) activation, and mitochondria depended on the apoptosis pathway in chondrocytes (Fig. 1E), as well as decreased collagen-2, the basis of cartilage tissues. Moreover, tBHP treatment, at different concentrations, promoted the synthesis and release of inflammatory factors, resulting in a dose-dependent degradation of cartilage tissues (Fig. 1F). According to these findings, tBHP triggered human chondrocytes to undergo apoptosis.

### 3.2 miR-34a-5p inhibits SIRT1 expression

**Fig. 2:**
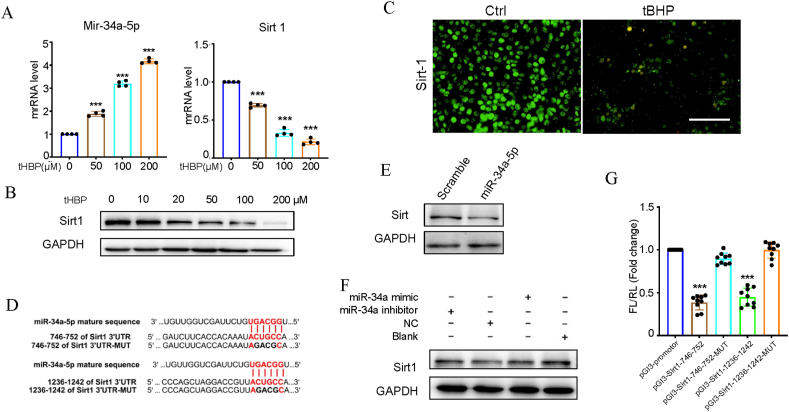
tBHP attenuated SIRT1 signaling in HC-OA cells via upregulation of miR-34a-5p. (A) The mRNA levels of miR-34a-5p and SIRT1 in the HC-OA cells exposed to different concentrations (0, 50, 100, 200 μM) of tBHP for 24 h were determined by quantitative real‐time PCR, and the results were normalized to GAPDH. Data are shown as the mean ± sem. (B) Protein expression of Sirt1 in the HC-OA cells exposed to different concentrations (0, 10, 20, 50, 100, 200 μM) of tBHP for 24 h was determined by Western blot analysis. (C) Representative immunofluorescence staining for Sirt1 in the HC-OA cells treated with PBS or 100 μM tBHP for 24 h. Scale bar represents 100 μm. (D) Predicted miR-34a-5p seed matches to the sequence in the 3ʹUTR of Sirt1 mRNA. The seed sequence of miR-34a-5p is CCGUCA, and the complementary nucleotide sequences are shown in red words. (E, F) Sirt1 protein expression in miR-34a-5p- and miR-34a-5p inhibitor-modified HC-OA cell Western blot assays. (G) Verification of Sirt1 as a target gene of miR-34a-5p by the dual luciferase reporter assay. Data are shown as the mean ± sem. *P < 0.05, **P < 0.01 and ***P < 0.001 vs. Cont. (For interpretation of the references to colour in this figure legend, the reader is referred to the Web version of this article.)

Our results revealed a dose-dependent effect of tBHP on SIRT1 and miR-34a-5p expression in HC-OA cells. Results of RT-qPCR, WB, and immunofluorescence assays revealed that miR-34-5p and Sirt1 are correlated in tBHP-induced apoptosis (Fig. 2A–C). Fig. 2D displays two binding sites for miR-34a-5p within the 3′-UTR of Sirt1. For the dual-luciferase assay, miR-34a-5p substantially diminished the activity of luciferase by binding to 764–752 and 1236–1242 sites on the 3′-UTR of Sirt1 (*p* < 0.05 and *p* < 0.01, respectively; Fig. 2E–G).

miR-34a-5p directly targets the SIRT1/p53 signaling pathway in human chondrocytes.

**Fig. 3:**
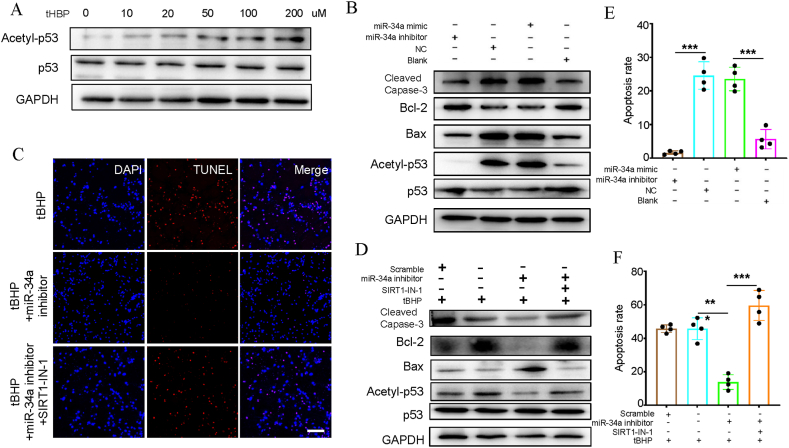
Activation of p53 upon tBHP stimulation is induced by miR-34a-5p-mediated Sirt1 downregulation. (A) Protein levels of p53 and its active form acetyl-p53 in the HC-OA cells exposed to PBS or different concentrations (0, 10, 20, 50, 100, 200 μM) of tBHP for 24 h were determined by Western blot analysis. (B) MicroRNA-34a (miR-34a-5p) directly targets the silent information regulator 1 (SIRT1)/p53 signaling pathway and promotes apoptosis in human chondrocytes. Western blot analysis of SIRT1, acetylated p53 (acetyl-p53), p53, Bax, Bcl-2 and cleaved caspase 3 protein expression. GAPDH was used as a loading control. (C) Representative images of immunostaining for apoptotic (TUNEL-positive, red) chondrocytes upon treatment with tBHP and miR-34a-5p inhibitor and/or 10 μM Sirt1 inhibitor SIRT1-IN-1. Nuclei were labeled with DAPI (blue). Scale bar represents 50 μm. (D) Western blot analysis of cleaved caspase-3, Bcl-2, Bax, acetyl-p53 and p53 upon treatment with tBHP, miR-34a-5p inhibitor and SIRT1-IN-1. The intensities of protein expression were quantified, normalized against the level of GAPDH and expressed as the fold change in protein abundance compared to the control. Data are the means ± sem of three independent experiment. (E) Effect of treatment with miR-34a-5p mimic or miR-34a-5p inhibitor on the chondrocyte apoptosis rate. Cell apoptosis rates were determined by Annexin V-fluorescein isothiocyanate/propidium iodide staining and analyzed by flow cytometry. Values are expressed as percentages of cell viability at Cont. Data are the means ± sem of five independent experiments in duplicate. *P < 0.05 and **P < 0.01 vs. Cont. (F) Effect of treatment with tBHP, miR-34a-5p inhibitor, and SIRT1-IN-1 on the chondrocyte apoptosis rate. Cell apoptosis rates were determined by Annexin V-fluorescein isothiocyanate/propidium iodide staining and analyzed by flow cytometry. Values are expressed as percentages of cell viability at Cont. Data are the means ± sem of five independent experiments in duplicate. *P < 0.05, **P < 0.01 and ***P < 0.001 vs. Cont. (For interpretation of the references to colour in this figure legend, the reader is referred to the Web version of this article.)

In HCT116 cells, according to earlier research, miR-34a-5p inhibits the SIRT1 expression in a direct manner [23]. The molecular function behind the biological processes of miR-34a-5p is clarified in this research. In line with WB assay findings, the acetyl-p53 protein level increased significantly in the group with miR-34a-5p in a tBHP dose-dependent manner. However, the total p53 level was unaffected (Fig. 3A). To determine whether SIRT1 protected HC-OA cells against tBHP-induced apoptosis via acetyl-p53, we treated 100 μM tBHP-treated HC-OA cells with a miR-34a-5p suppressor or miR-34a-5p-SIRT1 inhibitors (SIRT1-IN-1) at 10 μM. Results of TUNEL and WB assays showed that co-administration of the inhibitors led to an increase in the apoptotic nuclei and apoptosis-associated protein levels (Fig. 3B–D, F). Additionally, after miR-34a-5p excessive expression in human chondrocytes, the protein levels of pro-apoptotic Bcl-2 associated X-protein (Bax) and anti-apoptotic B-cell lymphoma-2 (Bcl-2), SIRT1/p53 pathway downstream genes, were inhibited and elevated, respectively (Fig. 3B–E).

Oxidative stress causes mitochondrial damage via the miR-34a-5p/Sirt1/p53 pathway.

**Fig. 4:**
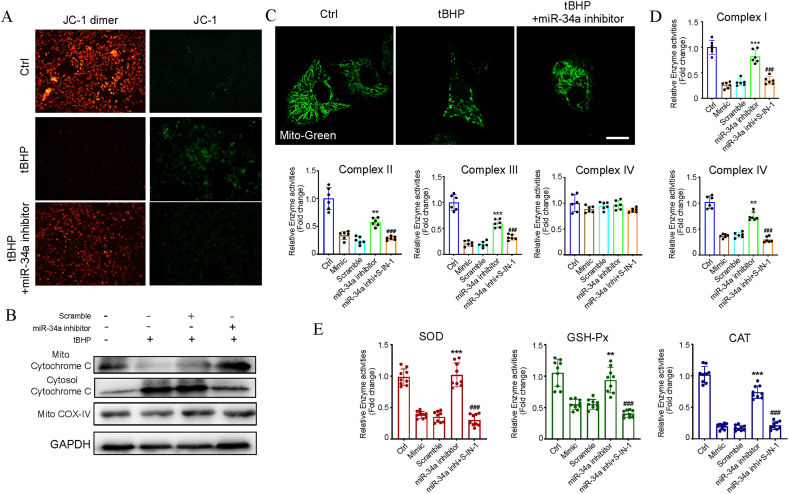
Disruption of the miR-34a-5p/Sirt1/p53 axis induced by tBHP caused oxidative stress-associated mitochondria. (A) The mitochondrial membrane potential (MMP) of the HC-OA cells treated with PBS, tBHP and miR-34a-5p inhibitor was determined with JC-1 using a confocal microscope. Representative images are shown (green, JC-1 monomer; red, JC-1 polymer). Scale bar represents 50 μm. (B) Western blotting for cytochrome C in the cytosolic and nuclear fractions of HC-OA cells treated with tBHP, a miR-34a-5p inhibitor. Intensities were quantified and normalized against the level of GAPDH or COX-IV and are expressed as fold changes of protein abundance relative to controls. (C) Mitochondrial morphology was determined by infecting HC-OA cells with an adenovirus vector carrying a foreign gene for mitochondrial complex IV (Ad-Mito-Green). Representative fluorescence images for GFP-labeled mitochondria within the cytosol. Scale bar represents 10 μm. (D) Activity of mitochondrial complex I, complex II, complex III, complex IV and complex V in the HC-OA cells treated with tBHP, miR-34a-5p inhibitor and/or SIRT1-IN-1 was measured by spectrophotometry. (E) The expression of genes encoding ROS scavengers (SOD, GSH-Px, CAT) was determined by an enzyme activity kit in HC-OA cells. *P < 0.05, **P < 0.01 and ***P < 0.001 vs. Cont. (For interpretation of the references to colour in this figure legend, the reader is referred to the Web version of this article.)

Given that mitochondrial dysfunction is primarily influenced by elevated oxidative stress, we examined if the miR-34a-5p/Sirt1/p53 pathway-related abnormal activation by ROS leads to mitochondrial impairment. Consistent with the *in vitro* findings, inhibiting miR-34a-5p post-tBHP treatment substantially restored the mitochondrial membrane potential (Fig. 4A). A heme-based ROS sensor found in mitochondria called cytochrome C peroxidase controls the antioxidant defense, and tBHP could induce cytochrome C (Cyto-C) release to the cytoplasm, leading to endothelial apoptosis. However, the miR-34a-5p inhibitor could protect the mitochondrial membrane, preventing the Cyto-C release into the cytosol (Fig. 4B). Additionally, biochemical assays revealed disruption of mitochondrial activity (mitochondrial complex I–IV's diminished activity; Fig. 4D) and confocal imaging revealed altered mitochondrial morphology (loss of mitochondrial cristae, vacuolization, and mitochondrial enlargement; Fig. 4C) in tBHP-treated cells. However, the miR-34a-5p suppressor significantly attenuated the tBHP effects on mitochondria function and structure (Fig. 4C and D). According to earlier research, oxidative damage to mitochondria is caused by the inhibition of ROS-scavenging enzymes including manganese superoxide dismutase (MnSOD) and glutathione peroxidase (GSH-Px). Moreover, catalase (CAT), GSH-Px, and superoxide dismutase (SOD) are downstream target genes of SIRT1. In accordance with our findings, inhibiting miR-34a-5p post-tBHP treatment substantially restored SOD, GSH-Px, and CAT activities in HC-OA cells (Fig. 4E).

### 3.3 SRT1720 attenuates ROS-mediated inflammation and apoptosis

**Fig. 5:**
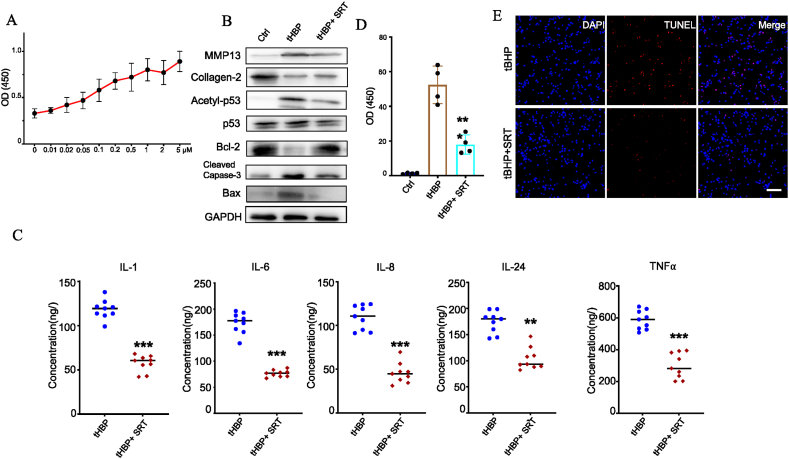
The Sirt1 agonist SRT 1720 attenuates tBHP-induced HC-OA cell injury. (A) HC-OA cells were cultured with different concentrations of SRT 1720 (0.01, 0.02, 0.05, 0.1, 0.2, 0.5, 1, 2, 5 μM) for 24 h. Cell viability was detected using CCK-8 assays. n = 6 wells per group. (B) Western blot analysis of cleaved caspase-3, Bcl-2, Bax, Collagen-2, Acetyl-p53, p53 and MMP13 upon treatment with 100 μM tBHP or 1 μM SRT 1720. (C) tBHP-mediated secretion of inflammatory factors in HC-OA cells. HC-OA cells were treated with 100 μM tBHP/1 μM SRT 1720 for 24 h. The concentrations of TNF-α, IL-1β, IL-6, IL-8 and IL-24 in cell supernatants were measured by ELISAs. (D) Effect of treatment with 100 μM tBHP/1 μM SRT 1720 for 24 h on the chondrocyte apoptosis rate. Cell apoptosis rates were determined by Annexin V-fluorescein isothiocyanate/propidium iodide staining and analyzed by flow cytometry. (E) Representative images of immunostaining for apoptotic (TUNEL-positive, red) chondrocytes. Nuclei were labeled with DAPI (blue). Scale bar represents 50 μm *P < 0.05, **P < 0.01 and ***P < 0.001 vs. Cont. (For interpretation of the references to colour in this figure legend, the reader is referred to the Web version of this article.)

HC-OA incubation in different concentrations of SRT1720 for 24 h revealed an increase in cell viability in a manner dependent on dose (Fig. 5A). In addition, WB analysis demonstrated that MMP-13, acetyl-p53, Bax, and cleaved caspase-3 were significantly decreased, whereas collagen-2 and Bcl-2 were substantially improved in the ROS-treated HC-OA cells as compared to control group (Fig. 5B). Moreover, ELISA results demonstrated that TNF-α, IL-1β, IL-6, IL-8, and IL-24 was significantly decreased in the SRT1720-treated HC-OA cells post-tBHP treatment compared with the control cells (Fig. 5C). Therefore, SRT1720 treatment significantly reversed the tBHP-induced effects in HC-OA cells and significantly altered the expression of the aforementioned proteins. These results indicate that SRT1720 may reduce inflammation and apoptosis in tBHP-treated HC-OA cells (Fig. 5B–D, E).

### 3.4 SRT1720 accelerates the surgery-induced OA progression in rats

**Fig. 6:**
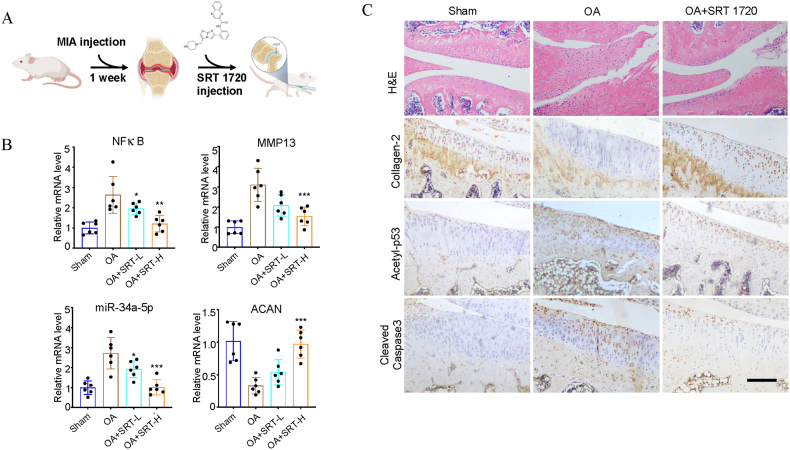
Activation of Sirt1 ameliorates surgery-induced cartilage degradation. (A) Schematic of the OA modeling and drug delivery in rats. (B) mRNA expressions of NFκB, MMP13, miR-34a-5p, and ACAN in rat knee cartilage were determined by quantitative real‐time PCR. Values are expressed as percentages of cell viability at Cont. Data are the means ± sem of six independent experiments in duplicate. *P < 0.05, **P < 0.01 and ***P < 0.001 vs. Cont. (C) Histological observation of sections stained with H&E and IHC staining of sections with collagen-2, acetyl-p53 and cleaved caspase 3 primary antibodies. Scale bar represents 200 μm.

Considering the results of the *in vitro* experiments, animal trials were performed for assessing the miR-34a-5p/Sirt1/p53 effects on surgically-induced OA. Rats' knee joints received injections of SRT1720 or saline (Fig. 6A), and the cartilage was collected for mRNA and histological assessment. The modified mRNA levels of nuclear factor (NF)-κB, MMP-13, miR-34a-5p, and aggrecan (ACAN) in the SRT1720-treated joints were substantially altered in comparison with the Sham group joints (Fig. 6B; *p* < 0.01). H&E staining revealed cartilage destruction in the OA group. A higher dose of SRT1720 given as intra-articular injection significantly attenuated MIA-induced cartilage damage in the OA-SRT1720 group, as indicated by immunohistochemical staining of cleaved caspase-3, collagen-2, and acetyl-p53 (Fig. 6C).

## 4 Discussion

OA causes progressive and irreversible degeneration of cartilaginous joints and serves as one of the key contributors to disability and injury in adults and older populations [24,25]. The miR-34a-5p′s function and treatment potential are elucidated in the current research and it is upregulated in ROS-triggered OA [26], together *in vivo* and *in vitro*. Suppression of miR-34a-5p in human HC-OA cells substantially downregulated the typical apoptotic [27] and catabolic markers' expression, as well as upregulated the key ECM markers' expression levels [28]. Since MIA increases the oxidative stress and reduces the antioxidant reactions, intra-articular MIA injection was employed to cause oxidative stress-triggered OA model in previous studies. The *in vivo* data also demonstrate that oral intake of SRT1720 alleviates MIA-induced cartilage tissue injury in rat knees [29].Based on this, we used oxidative stress to establish cellular and animal models related to OA in this study, further verifying the significance of miR-34a-5p targeting SIRT1/p53 signaling axis in influencing inflammatory processes and repairing chondrocyte damage. We also preliminarily elucidated the mechanism of miR-34a-5p/SIRT1/p53 axis in oxidative stress-induced OA progression.

Numerous studies have revealed important functions for miRNAs in the regulation of ECM, chondrogenesis, inflammatory reactions, and other biological mechanisms that control typical joint function and uphold homeostasis. For example, miR-140 promotes mesenchymal stem cell chondrogenesis and stimulates cartilage injury in a rat knee OA model; miR-146a and miR-146b appear to be key miRNAs in the inflammatory response in OA; miR-149 targets the mRNAs of TNFα, IL-1β, and IL-6, and its downregulation might contribute to OA progression [2,30,31]; and inhibition of miR-34a-5p expression attenuates synovial inflammation and cartilage damage in cruciate ligament removed rat knee joints [32].

One of the main factors accelerating OA is oxidative stress [33], and ROS, including hydrogen peroxide, superoxide anion, and hydroxyl radicals, are toxic by-products of normal cellular metabolism. The ability of cells to counteract ROS toxicity and displace the ROS molecules diminishes over time, and their accumulation can be detected in aging organs. Being the primary source of ROS in cells, mitochondria appeared to be a major target of ROS accumulation during aging. tBHP oxidizes the intracellular proteins and lipids in the HC-OA cells and triggers ROS production, while MIA inhibits glyceraldehyde-3-phosphate dehydrogenase activity, reduces pentose phosphate pathway flux, subsequently reducing NAPDH and NADH production, resulting in a decreased cellular resistance to oxidative stress, ultimately instigating apoptosis. In OA chondrocytes, the oxidant/antioxidant balance collapse results in an altered redox state that facilitates cartilage degradation [34] and renders cells more vulnerable to oxidant-mediated apoptosis, leading to OA injuries that happen under arthritic conditions [35]. Previous studies demonstrated that oxidative stress stimulates the miR-34a-5p expression in human cardiac and carcinoma cell lines [3]. Endogenous ROS are balanced and quenched by intrinsic antioxidant defense systems involving enzymes, including SOD, thioredoxin reductase (TrxRs), CAT, and GSH-Px [36]. In addition, antioxidant supplementation (vitamins C and E) prevents inflammatory arthritis and maintains healthy joints in OA patients.

According to this study's findings, ROS-induced upregulation of miR-34a-5p inhibited SIRT1's expression in HC-OA cells, resulting in the inhibition of acetyl-p53 deacetylation, thereby triggering p53-mediated apoptosis. In addition, SIRT1 can cause deacetylation of acetyl-nuclear erythroid factor 2-related factor 2 (Nrf2), which is a significant transcription factor for the anti-inflammatory as well as antioxidant responses involved in antioxidant enzymes' expression regulation [1]. These effects are ultimately manifested in decreased apoptosis rate, increased expression of antioxidant enzymes, and consequent increase in mitochondrial activity under tBHP-stimulated conditions [37]. Our study also showed that p53 positively regulates the miR-34a-5p′s expression levels, leading to a positive amplification of p53-induced apoptotic effects [13]. Downregulation of SIRT1 expression correlated with downregulation of collagen-2 and aggrecan (ACAN) and upregulation of matrix metalloproteinases, highlighting the presence of a positive feedback loop in the miR-34a-5p/SIRT1/p53 axis [38].

Moreover, SRT1720 [39] alleviated the process of articular cartilage damage and protected against OA in the rat OA model. The results of SIRT1 and p53 expression in rat knee joints, detected by RT-qPCR and immunohistochemical staining, indicated that SRT1720 at different doses could inhibit the miR-34a-5p′s expression, NF-κB, and MMP13; prevent the caspase-3's cleavage; and upregulate the expression of Col-2a and ACAN, alleviating pathological changes in the OA model [40]. Gene expression analysis revealed that the protective effect of SRT170 was greater in the high-dose groups in comparison with the low-dose groups, implying a dose-dependent SRT1720 effect [24]. Additionally, it was further confirmed that SRT1720 played a crucial role in the prevention of chondrocyte apoptosis by activating SIRT1 expression. Intra-articular injection of SRT1720 had a significant effect on OA progression; however, it will be necessary to test efficacy using oral or other drug delivery systems, such as gelatin hydrogel delivery of drug micelles, for future practical applications.

The limitations of our study are as follows: firstly, younger rats were used for the *in vivo* experimentation, although OA is prevalent in aging populations. Secondly, MIA-induced ROS production is rapid and severe *in vivo*, and our surgically-induced OA model may not truly reflect the progression of OA in humans. Therefore, it is necessary to assess the effectiveness and safety of SRT1720 administration in different OA models. Lastly, although the full pharmacological action of SRT1720 is unknown and there is no clinical evidence that SRT1720 is also effective in humans, our findings suggest that systemic administration of SRT1720 can slow down OA progression.

## 5 Conclusion

According to our findings, SIRT1 plays a mediating role in miR-34a-5p-related apoptotic activity in ROS-induced OA, and elevated levels of miR-34a-5p can cause joint degeneration, leading to OA progression. Therefore, preventing the miR-34a-5p′s expression or activating SIRT1 can be a candidate treatment target for OA treatment.

## Ethics approval and consent to participate

All surgical procedures were performed following the instructions approved by the Institutional Animal Care and The Animal Ethical and Welfare Committee of Peking University, Shenzhen Hospital (Permit Number: 2020-301).

## Funding

This research was supported by grants from 10.13039/501100001809National Natural Science Foundation of China (No. 82172432; No. 82102568), Guangdong Province Medical Science and Technology Research Foundation Project (No. A2019257), Shenzhen Key Medical Discipline Construction Fund (No. SZXK023), Shenzhen “San-Ming” Project of Medicine (No.SZSM202211038), Shenzhen Science and Technology Program (No.JCYJ20220818102815033; No.KCXFZ20201221173411031; No.JCYJ20210324110214040), Guangdong Basic and Applied Basic Research Foundation (No.2022B1515120046; No.2022A1515220111; No. 2021A1515012586), the Scientific Research Foundation of PEKING UNIVERSITY SHENZHEN HOSPITAL (No. KYQD2021099) and Shenzhen High-level Hospital Construction Fund.

## Consent for publication

The manuscript has not been previously published and is not being concurrently submitted elsewhere.

## Availability of data and material

Data are available from the corresponding authors upon reasonable request with the permission of Department of Bone and Joint in Peking University Shenzhen Hospital.

## Data availability statement

The data that support the findings of this study are available from the corresponding author, [Ming Lei], upon reasonable request.

## CRediT authorship contribution statement

**Meng Zhou:** Writing – original draft, Software, Resources, Methodology, Formal analysis, Data curation, Conceptualization. **Bi Liu:** Validation, Software, Resources, Data curation. **Hai-Ming Ye:** Resources, Formal analysis, Data curation. **Jia-Ning Hou:** Software, Formal analysis, Data curation. **Yi-Cong Huang:** Software, Formal analysis, Data curation. **Peng Zhang:** Software, Formal analysis, Data curation. **Liang Gao:** Validation, Supervision, Methodology. **Hao-Tian Qin:** Methodology, Formal analysis, Data curation. **Yi-Fei Yang:** Software, Resources, Data curation. **Hui Zeng:** Visualization, Validation, Supervision, Project administration, Methodology, Funding acquisition. **Bin Kang:** Visualization, Validation, Supervision, Software, Resources, Methodology, Data curation. **Fei Yu:** Writing – review & editing, Visualization, Validation, Supervision, Project administration, Methodology, Investigation, Funding acquisition, Conceptualization. **De-Li Wang:** Visualization, Validation, Supervision, Methodology, Investigation, Funding acquisition. **Ming Lei:** Writing – review & editing, Visualization, Validation, Supervision, Methodology, Investigation, Conceptualization.

## Declaration of competing interest

The data results of this study can be obtained from the corresponding author according to reasonable requirements. There is no conflict of interest between the authors.

## Abbreviations

OA: osteoarthritis
ELISA: enzyme-linked immunosorbent assay
tBHP: tert-Butyl hydroperoxide
ACAN: Aggrecan
TUNEL: terminal deoxynucleotidyl transferase dUTP nick end labeling
CAT: catalase
qPCR: quantitative polymerase chain reaction
MMP-13: matrix metallopeptidase-13
SPF: specific-pathogen-free
SOD: superoxide dismutase
MIA: monosodium Iodoacetate and DAPI: 4′, 6-diamidino-2-phenylindole
