# Supplementary material for: ROS-induced imbalance of the miR-34a-5p/SIRT1/p53 axis triggers chronic chondrocyte injury and inflammation
Source: Heliyon. 2024 May 22;10(11):e31654. doi: 10.1016/j.heliyon.2024.e31654 (PMC11140697; doi:10.1016/j.heliyon.2024.e31654)
Supplement: Multimedia component 1 [file mmc1.docx]

**Figure 1.**

**(E)** tBHP Concentration treatment on HC-OA cells.


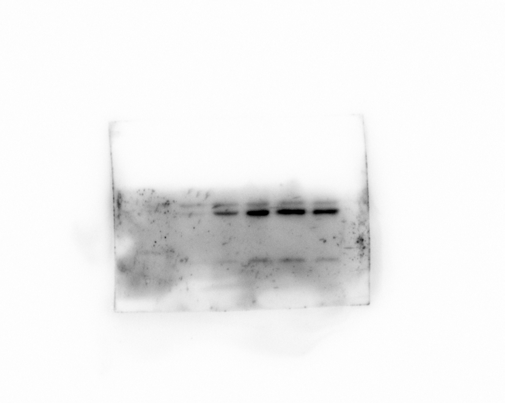

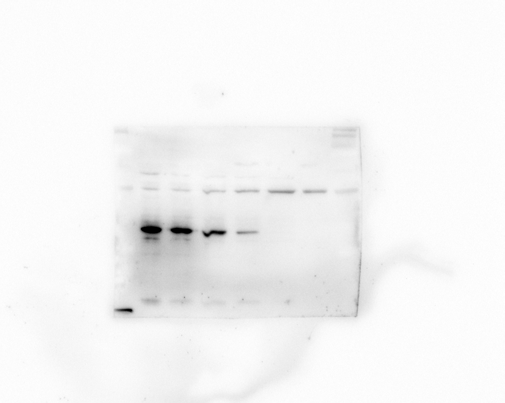


Cleaved caspase-3 Bcl-2


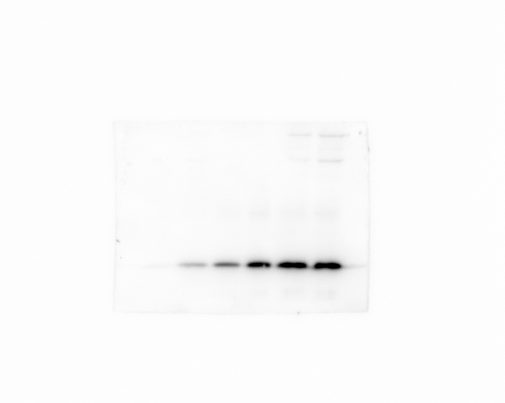

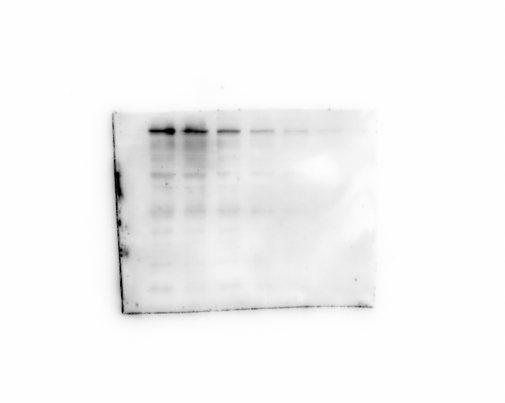


Bax Collagen-2


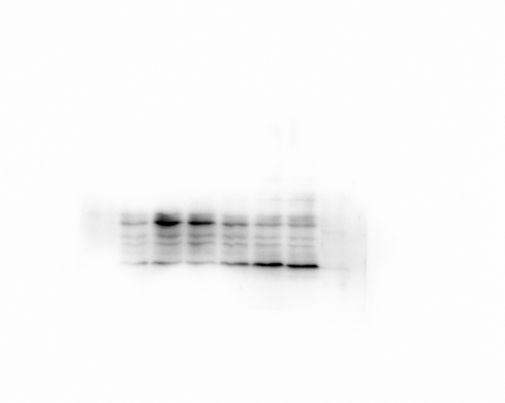

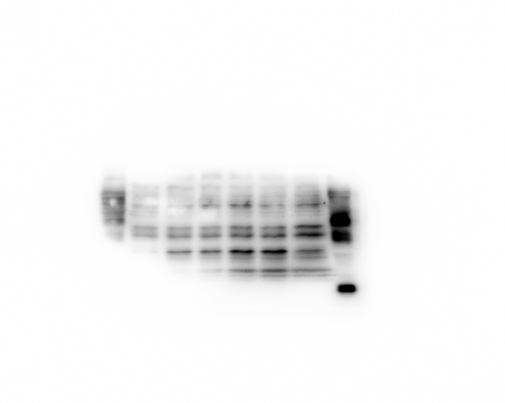


IL-1β MMP13


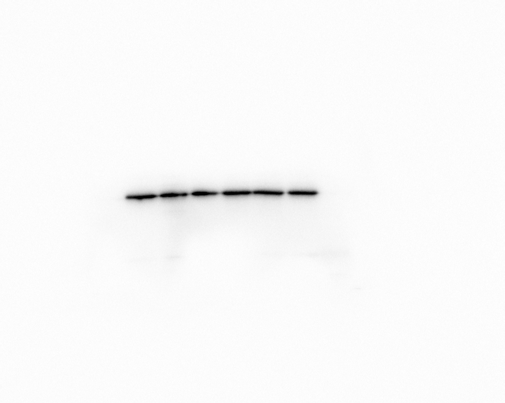


GAPDH

**(E)** tBHP in 100μM concentration treatment on HC-OA cells.


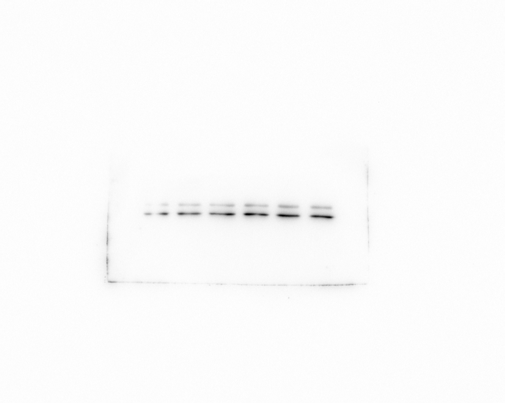

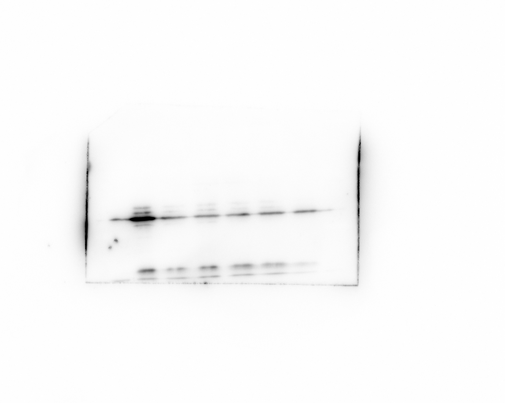


Cleaved caspase-3 Bcl-2


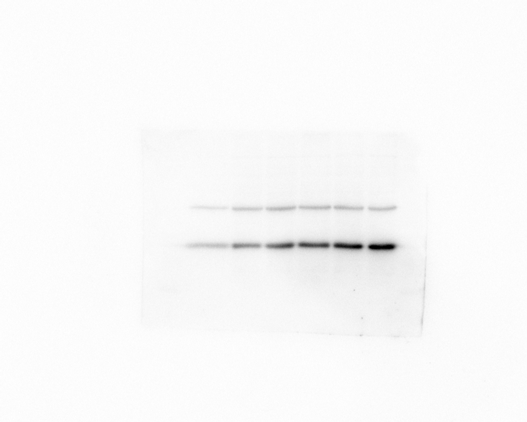

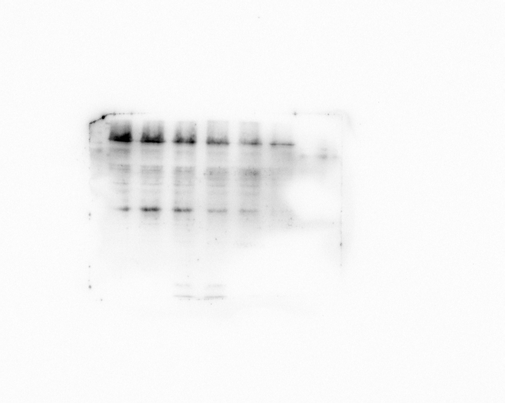


Bax Collagen-2


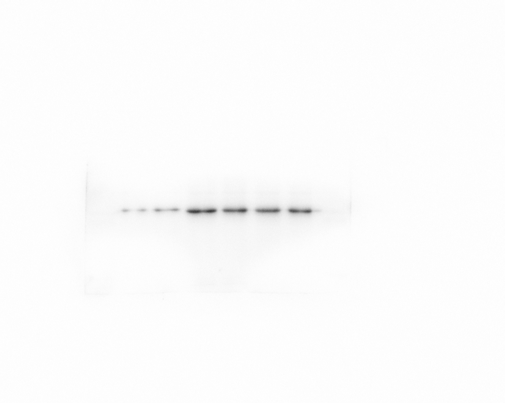

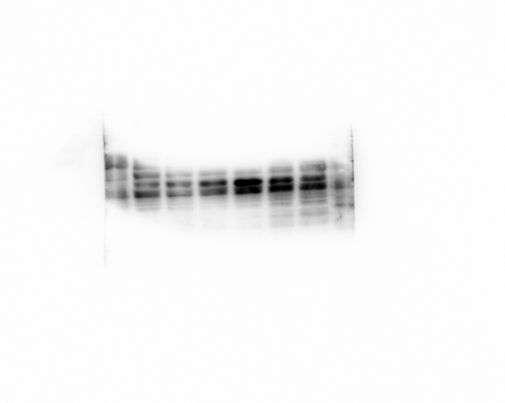


IL-1β MMP13


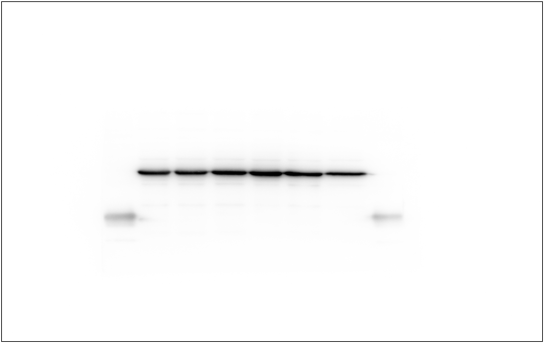


GAPDH

(E) Western blot analysis for cleaved caspase-3, Bcl-2, Bax, Collagen-2, IL-1β and MMP13.

**Figure 2.**

**(B)**


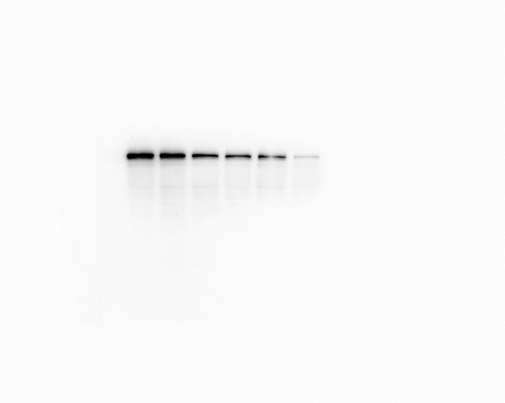

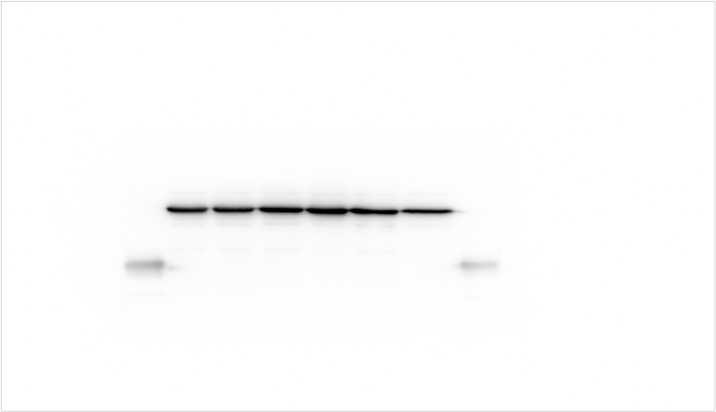


Sirt1 GAPDH

(B) Protein expression of Sirt1 in the HC-OA cells exposed to different concentrations (0, 10, 20, 50, 100, 200 μM) of tBHP for 24 h was determined by Western blot analysis.

**(E)**


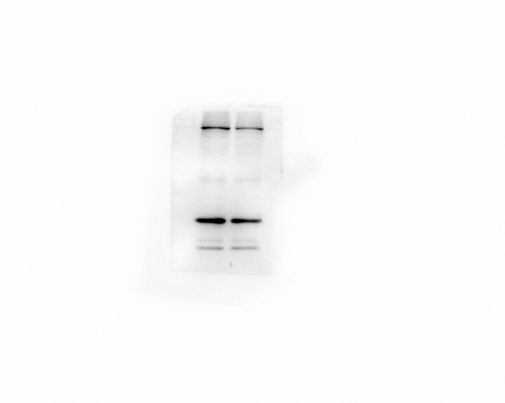

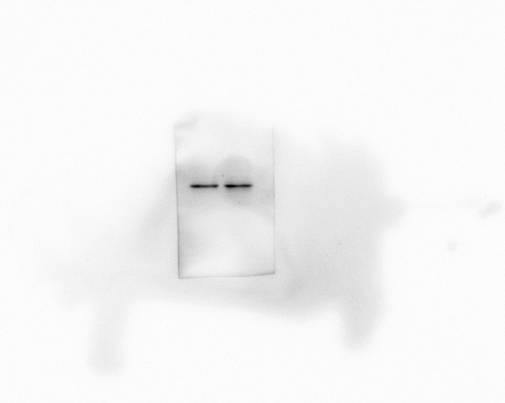


Sirt1 GAPDH

(E) Sirt1 protein expression in miR-34a-5P mimiced HC-OA cell Western blot assays.

**(F)**


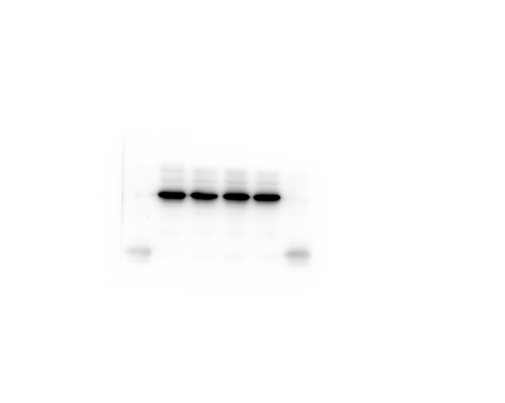

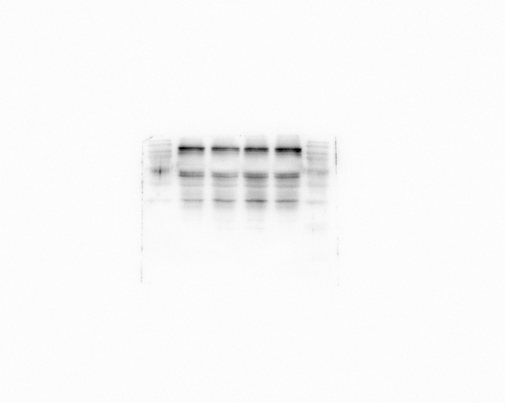


Sirt1 GAPDH

(F) Sirt1 protein expression in miR-34a-5p- and miR-34a-5p inhibitor-modified HC-OA cell Western blot assays.

**Figure 3.**

**(A)**

**
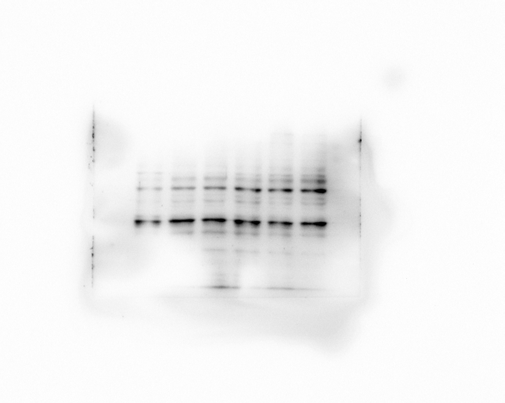

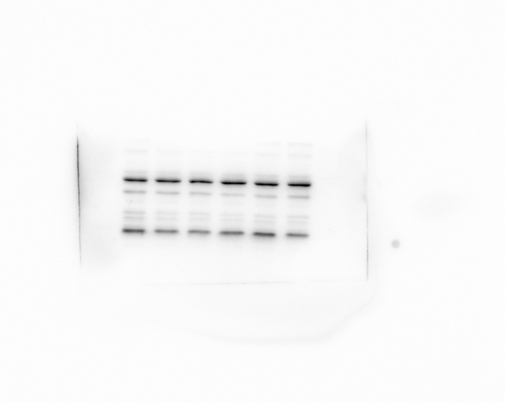
**

Acetyl-p53 p53


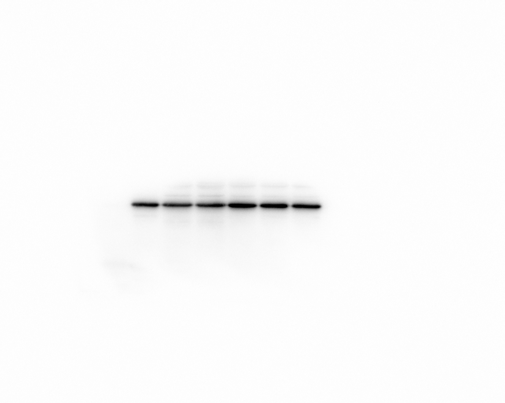


GAPDH

(A) Protein levels of p53 and its active form acetyl-p53 in the HC-OA cells exposed to PBS or different concentrations (0, 10, 20, 50, 100, 200 μM) of tBHP for 24 h were determined by Western blot analysis.

**(B)**


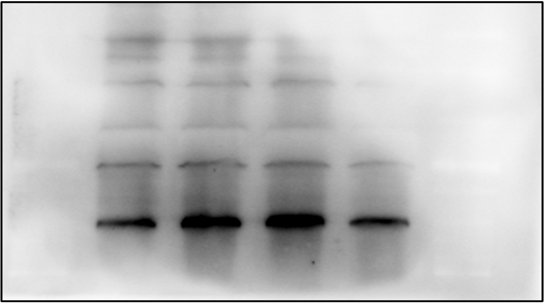

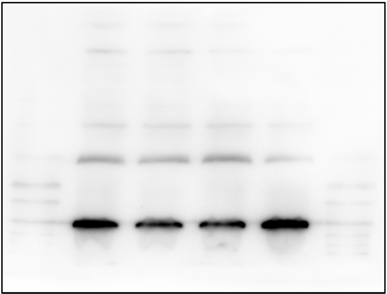


Cleaved caspase-3 Bcl-2


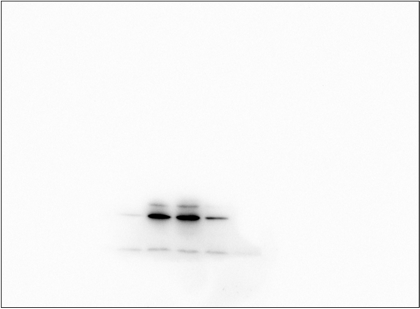

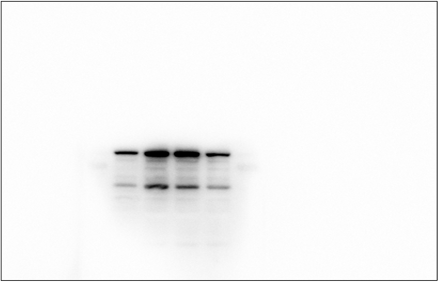


Bax Acetyl-p53

**
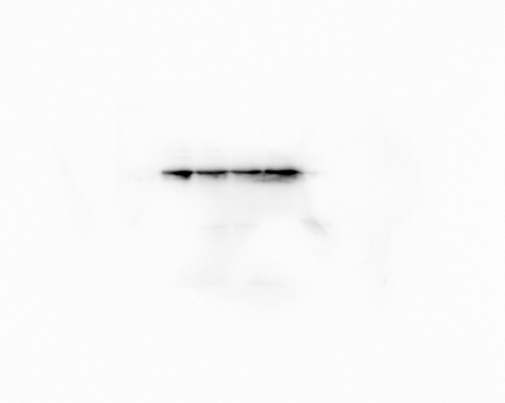

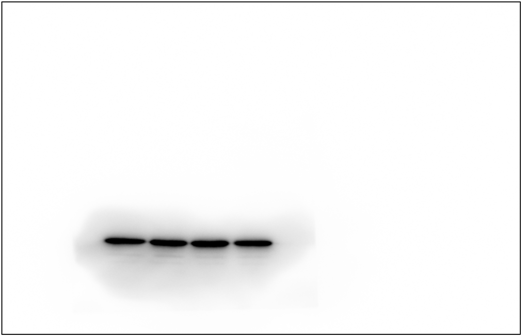
**

p53 GAPDH

(B) MicroRNA-34a (miR-34a-5p) directly targets the silent information regulator 1 (SIRT1)/p53 signaling pathway and promotes apoptosis in human chondrocytes. Western blot analysis of SIRT1, acetylated p53 (acetyl-p53), p53, Bax, Bcl-2 and cleaved caspase 3 protein expression. GAPDH was used as a loading control.

**(D)**

**
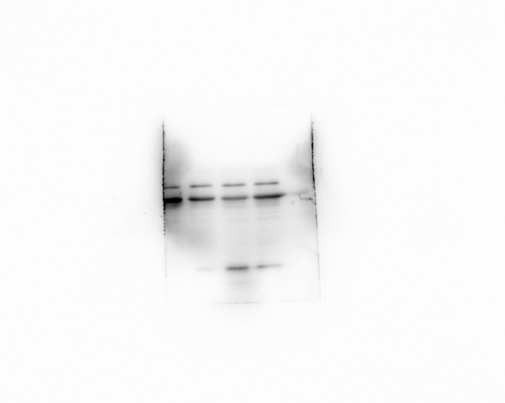

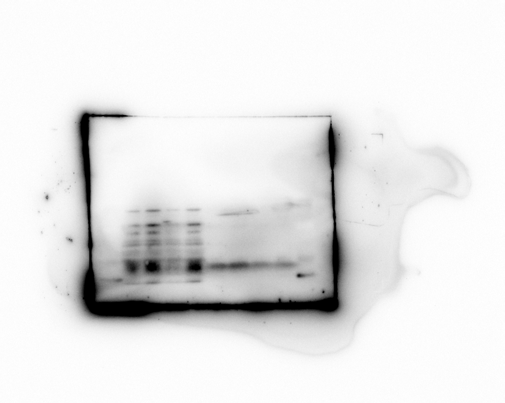
**

Cleaved caspase-3 Bax

**
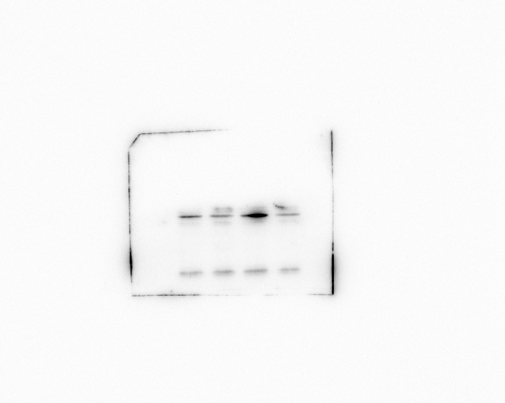

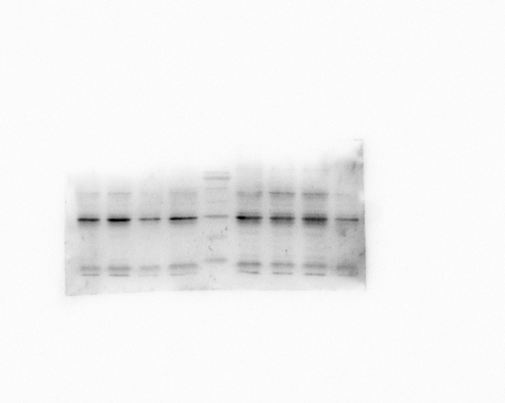
**

Bcl-2 Acetyl p53


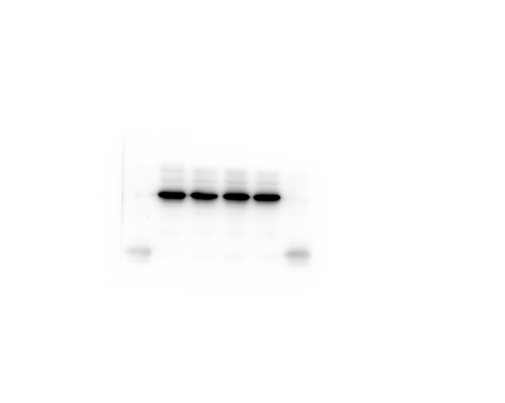
 **
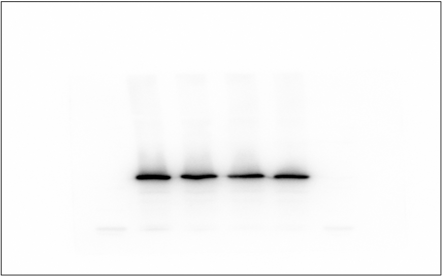
**

p53 GAPDH

(D) Western blot analysis of cleaved caspase-3, Bcl-2, Bax, acetyl-p53 and p53 upon treatment with tBHP, miR-34a-5p inhibitor and SIRT1-IN-1. The intensities of protein expression were quantified, normalized against the level of GAPDH and expressed as the fold change in protein abundance compared to the control. Data are the means±sem of three independent experiment.

**Figure 4.**

**(B)**

**
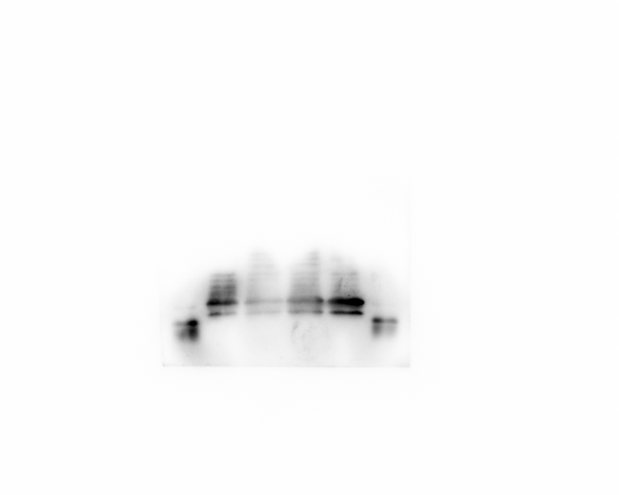

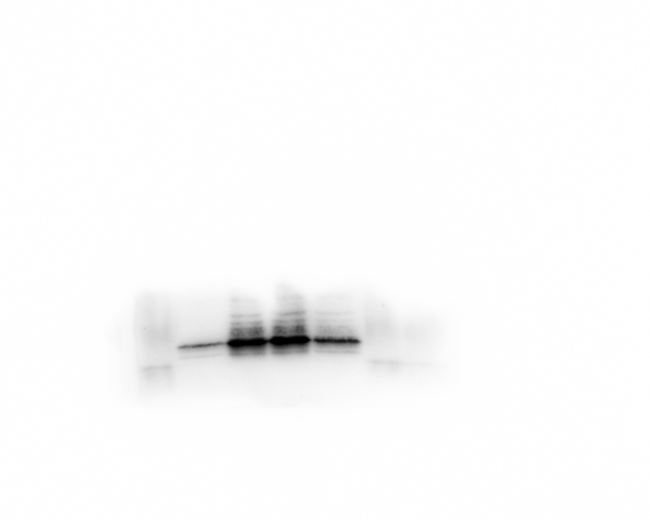
**

Mito-cytochrome C Cytochrome C


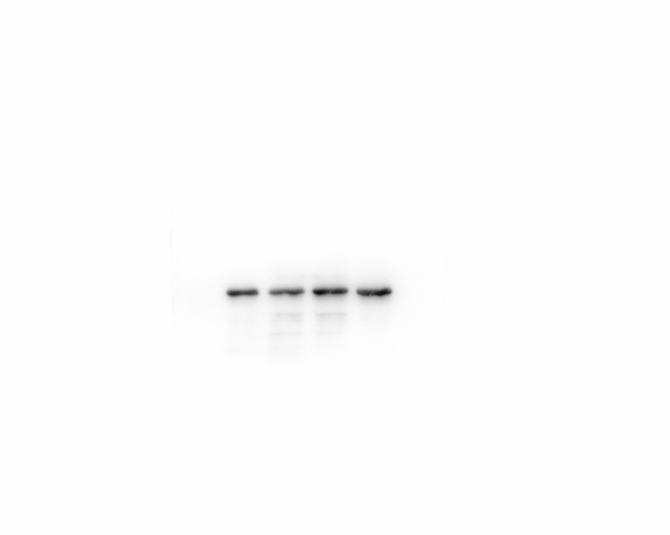
 **
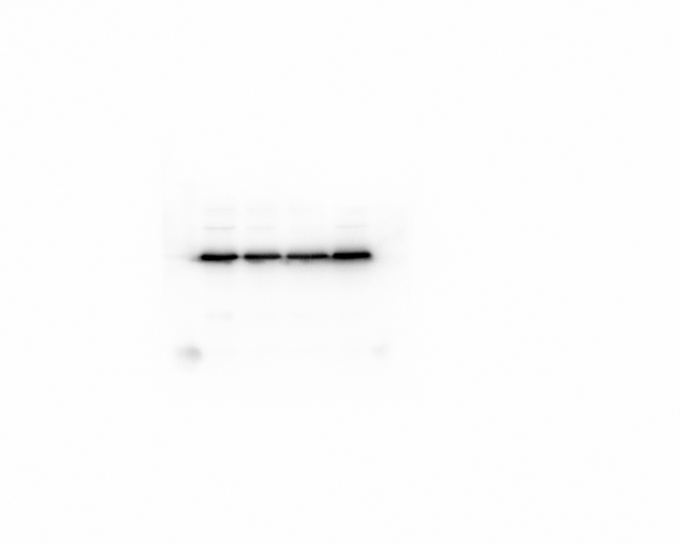
**

Mito COX-IV GAPDH

(B) Western blotting for cytochrome C in the cytosolic and nuclear fractions of HC-OA cells treated with tBHP, a miR-34a-5p inhibitor. Intensities were quantified and normalized against the level of GAPDH or COX-IV and are expressed as fold changes of protein abundance relative to controls.

**Figure 5.**

**(B)**

**
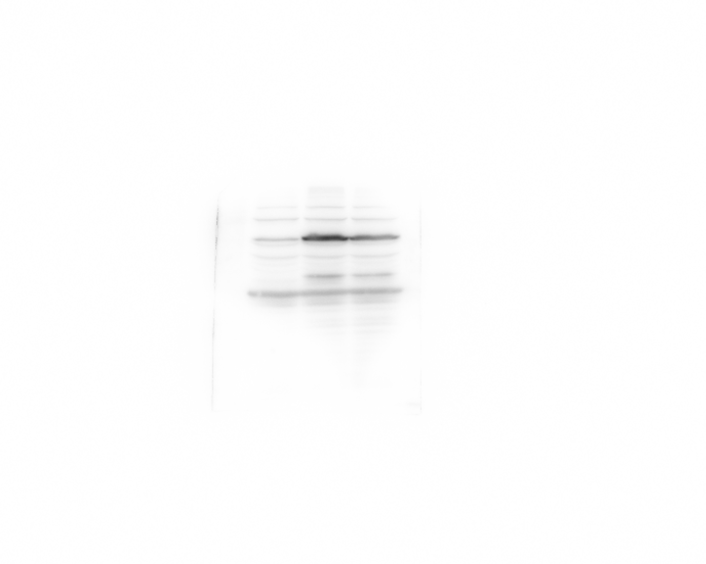

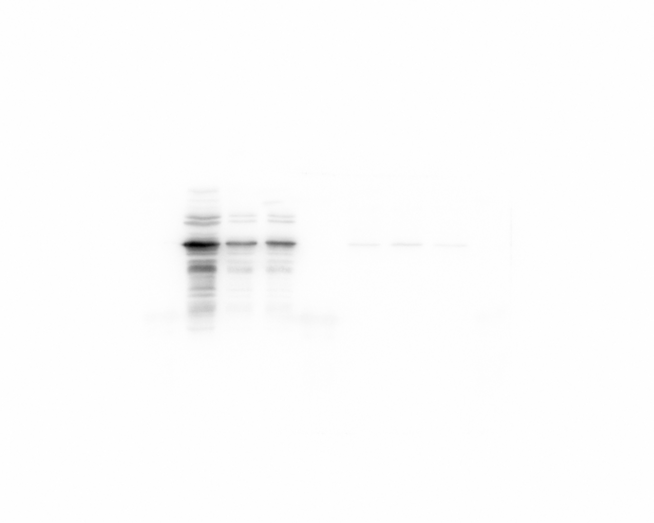
**

MMP13 Collagen-2


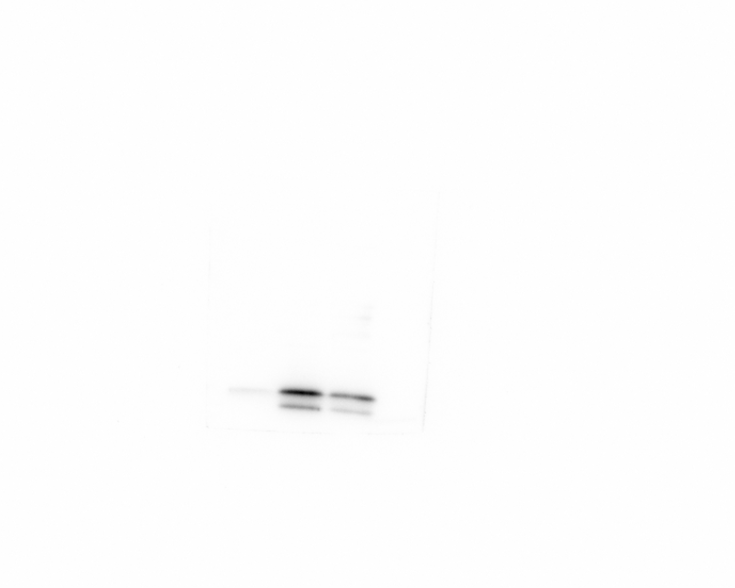
 **
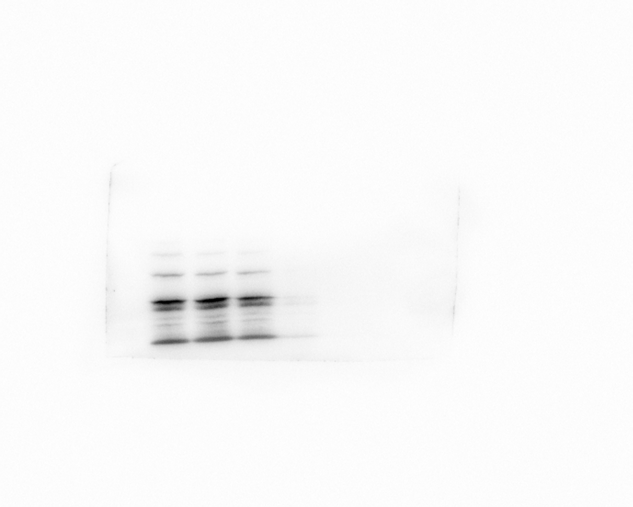
**

Acetyl p53 p53


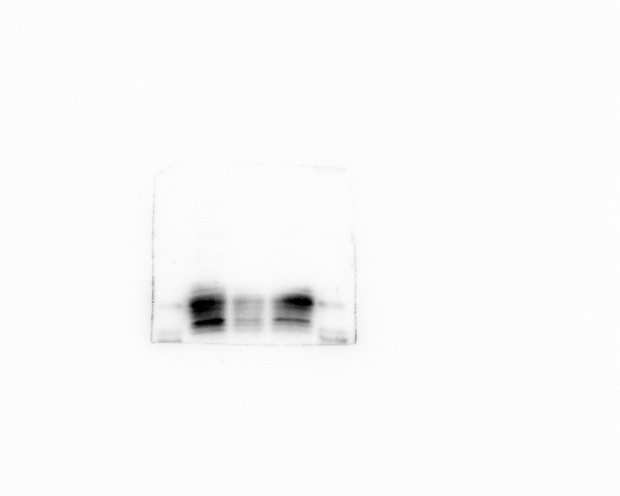
**
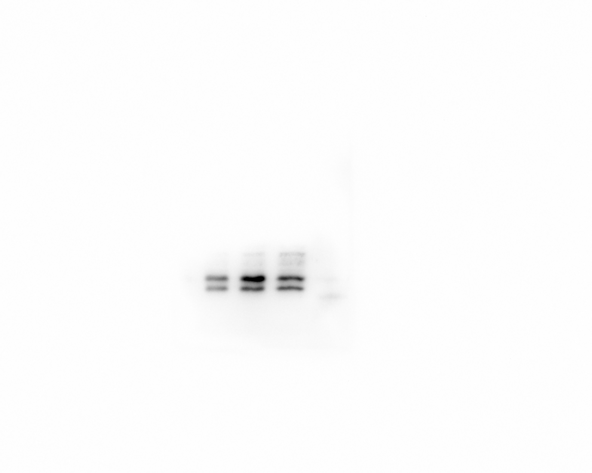
**

Bcl-2 Cleaved caspase-3


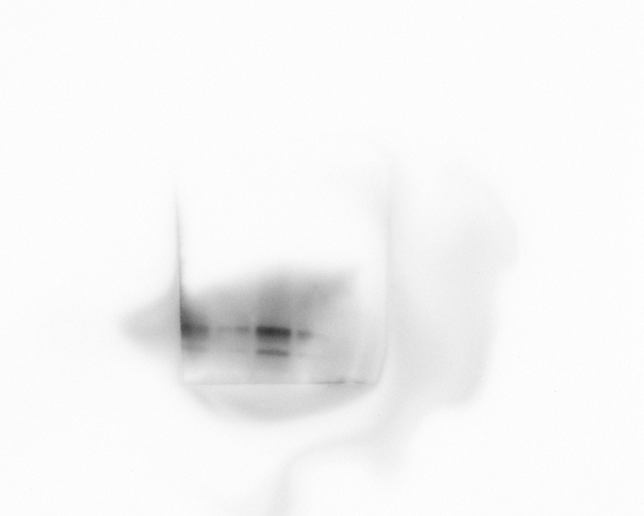

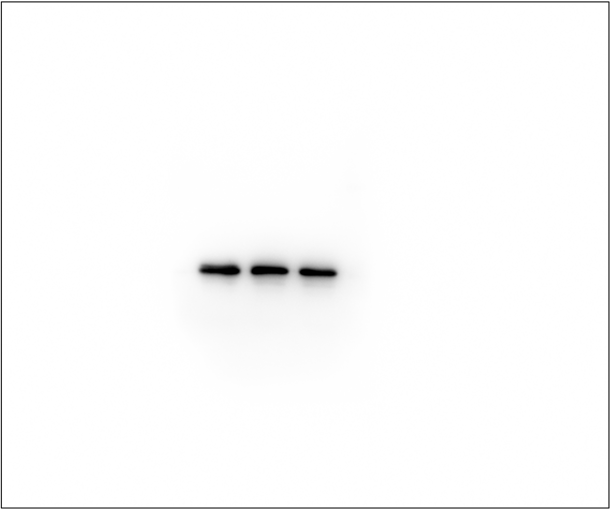


Bax GAPDH

**(B) Western blot analysis of cleaved caspase-3, Bcl-2, Bax, Collagen-2, Acetyl-p53, p53 and MMP13 upon treatment with 100 μM tBHP or 1 μM SRT 1720.**
